# Correlation of microRNA‐125a/b with acute respiratory distress syndrome risk and prognosis in sepsis patients

**DOI:** 10.1002/jcla.23098

**Published:** 2020-01-22

**Authors:** Shilei Li, Danna Zhao, Jie Cui, Lizeng Wang, Xiaohua Ma, Yong Li

**Affiliations:** ^1^ Emergency Department Cangzhou Central Hospital Cangzhou China; ^2^ Laboratory Department Cangzhou People Hospital Cangzhou China

**Keywords:** acute respiratory distress syndrome, microRNA‐125a, microRNA‐125b, sepsis

## Abstract

**Objective:**

This study was conducted to explore the association of microRNA (miR)‐125a and miR‐125b with acute respiratory distress syndrome (ARDS) risk and to investigate their correlation with clinical characteristics and prognosis in sepsis patients.

**Methods:**

Totally 150 sepsis patients admitted to our hospital were consecutively enrolled and another 150 healthy subjects were enrolled as healthy controls (HCs). Their blood samples were collected for miR‐125a and miR‐125b detection by real‐time quantitative polymerase chain reaction. Besides, ARDS occurrence and 28‐day mortality were documented in all sepsis patients.

**Results:**

MiR‐125a and miR‐125b relative expressions were increased in ARDS‐sepsis patients/non‐ARDS‐sepsis patients compared with HCs, while only miR‐125b but not miR‐125a was elevated in ARDS‐sepsis patients compared with non‐ARDS‐sepsis patients. Receiver operating characteristic (ROC) curve presented that miR‐125a (AUC: 0.650, 95%CI: 0.549‐0.750) and miR‐125b (AUC: 0.739, 95%CI: 0.653‐0.823) could differentiate ARDS‐sepsis patients from non‐ARDS‐sepsis patients, and miR‐125b was of increased predictive value compared with miR‐125a numerically. In sepsis patients, miR‐125a relative expression was positively associated with serum creatinine (Scr), chronic health evaluation (APACHE) II score, sequential organ failure assessment (SOFA) score, and miR‐125b was positively associated with Scr, C‐reactive protein (CRP), APACHE II score, SOFA score, and chronic obstructive pulmonary disease. All sepsis patients were categorized into survivors and deaths according to 28‐day mortality, and miR‐125b but not miR‐125a was upregulated in deaths compared with survivors.

**Conclusion:**

Both of miR‐125a and miR‐125b predict ARDS risk, while only miR‐125b is of value in prognosis prediction in sepsis patients.

## 1 INTRODUCTION

Sepsis is defined as a dysregulated host response to infection which is correlated with a life‐threatening organ dysfunction.^1^ As a major public health concern, sepsis accounts for considerable healthcare costs and serves as a leading cause of critical illness and mortality globally with an increasing incidence and in‐hospital mortality rate of 15%‐25%.^2^, ^3^ The effective treatments for sepsis include timely removal of the source of infection and appropriate antimicrobial therapy; however, the prognosis of most sepsis patients is still unfavorable.^4^ Acute respiratory distress syndrome (ARDS), as the most common and serious complication of sepsis, is a pulmonary dysfunction due to various causes, such as systemic inflammation and uncontrolled cytokine, leading to increased mortality in sepsis patients.^5^ Therefore, it is essential to explore the biomarkers for predicting ARDS risk and monitoring the prognosis in sepsis patients.

MicroRNAs (miRNA) are universal non‐coding RNAs that regulate the expressions of target genes via inhibiting the translation or depredation of mRNA and accumulating evidences indicate that miRNAs are involved in the regulation of the immune response and inflammation.^6^, ^7^ MiR‐125a and miR‐125b, which belong to miR‐125 family, have been reported to be involved in various processes of immune responses and inflammation.^8^, ^9^ For instance, miR‐125a high expression is reported to contribute to the upregulation of inflammatory cytokines and chemokines, such as IL‐β, IL‐6, and TNF‐α, in lupus nephritis.^10^ As for miR‐125b, its overexpression elevated the expressions of proinflammatory factors including TNF‐α, IL‐6, IL‐1β, and p‐p65 via activating NF‐κB pathway, meanwhile its involvement in the immune response and inflammation is reported in acute lung injury.^11^, ^12^ In addition, the regulatory roles of miR‐125a as well as miR‐125b in reactive oxygen species (ROS) level, which is associated with oxygen and glucose deprivation, are reported in several organs including lung.^13^, ^14^ Considering the association of miR‐125a and miR‐125b with proinflammatory factors in immune, inflammation responses and their regulating roles in lung injuries, we hypothesized that they might be of value in predicting ARDS risk as well as prognosis in sepsis patients. However, no related research has been conducted before. Thus, we performed this study to explore the association of miR‐125a and miR‐125b with ARDS risk and investigated its correlation with clinical characteristics as well as the prognosis in sepsis patients.

## 2 MATERIALS AND METHODS

### 2.1 Participants

In our emergency department, there were 85 beds (including 10 beds of respiratory intensive care unit), and we had a medical center with various high‐tech treatments including mechanical ventilation and respiratory mechanics monitoring, hemodynamic monitoring and treatment, blood purification, and severe ultrasound. The inclusion criteria were as follows: (a) diagnosed as sepsis in line with the Third International Consensus Definitions for Sepsis and Septic Shock (Sepsis‐3);^15^ (b) age ≥18 years; (c) no history of cancers or haematological malignancies. The exclusion criteria included the following: (a) died within 24 hours after admission; (b) infected with human immunodeficiency virus; (c) received immunosuppressive therapy within 6 months; (d) pregnant or lactating women. In addition, 150 healthy subjects who underwent physical examination in our hospital were enrolled as healthy controls (HCs). The screen criteria of HCs were as follows: (a) age above 18 years old; (b) had no history of sepsis or malignancies; (c) had no obvious abnormalities confirmed by physical examination; (d) not pregnant or lactating women. This study was approved by the Ethics Committee of our hospital. All participants or their guardians provided written informed consents before enrollment.

### 2.2 Clinical data collection

Clinical characteristics of sepsis patients were documented after enrollment, including age, gender, body mass index (BMI), smoke status, and chronic comorbidities (such as chronic obstructive pulmonary disease [COPD], cardiomyopathy, chronic kidney failure, and cirrhosis). The laboratory indexes measured immediately after admission, such as serum creatinine (Scr), albumin, white blood cell (WBC), and C‐reactive protein (CRP), were also recorded. Besides, the acute physiology and chronic health evaluation (APACHE) II score and the sequential organ failure assessment (SOFA) score were documented as well, which were evaluated within 24 hours after admission and used to assess severity of sepsis and severity of organ dysfunction.

### 2.3 Sample collection

Blood samples of all sepsis patients were collected within 24 hours after admission, and the blood samples of HCs were collected on the enrollment. All blood samples were centrifuged at 1600 g for 10 minutes at 4°C. Subsequently, the supernatants were separated into Eppendorf tubes and further centrifuged at 12 000 g for 10 minutes at 4°C. Finally, the plasma was obtained and stored at −80°C until determination.

### 2.4 MiR‐125a and miR‐125b detection

The relative expressions of miR‐125a and miR‐125b in plasma were detected by real‐time quantitative polymerase chain reaction (qPCR). Total RNA was extracted from plasma using PureZOL RNA isolation reagent (Bio‐Rad). Reverse transcription of cDNA was performed using RT‐PCR Quick Master Mix (Toyobo). Then, qPCR was performed using SYBR Green Real time PCR Master Mix (Toyobo) to quantify miR‐125a and miR‐125b expressions. The result was calculated using 2^−ΔΔCt^ method, and all expressions were normalized to U6. The primers used in qPCR were listed as follows:

miR‐125a, forward primer: ACACTCCAGCTGGGTCCCTGAGACCCTTTAAC, reverse primer: TGTCGTGGAGTCGGCAATTC. miR‐125b, forward primer: ACACTCCAGCTGGGTCCCTGAGACCCTAACTT; reverse primer: TGTCGTGGAGTCGGCAATTC. U6, forward primer: CTCGCTTCGGCAGCACATATACTA, reverse primer: ACGAATTTGCGTGTCATCCTTGC.

### 2.5 Treatment and follow‐up

Standard treatments and resuscitation were administered to patients after the diagnosis was established, which were performed as recommended by the International Guidelines for Management of Sepsis and Septic Shock.^16^ All patients were followed up for 28 days, and the patients who died during follow‐up were recorded for evaluation of 28‐days mortality. All patients were categorized into survivors and deaths according to the survival status during 28‐days follow‐up.

### 2.6 Acute respiratory distress syndrome (ARDS) assessment

During the hospitalization, close surveillance was conducted for all patients, especially for the patients with risk factors such as pneumonia, inhalational injury, pulmonary contusion, and so on. The patients who developed ARDS were documented for assessment of ARDS incidence. The ARDS was confirmed on the basis of onset time, chest imaging (chest X‐ray or CT scan) and the origin of edema, which was in accordance with the 2012 Berlin ARDS definition.^17^ According to whether ARDS occurred or not, all sepsis patients were categorized as ARDS‐sepsis patients and non‐ARDS‐sepsis patients.

### 2.7 Statistical analysis

Continuous variables were checked for normality by using quantile‐quantile plot (Q‐Q plot). The normally or approximately normal distributed variables were presented as mean ± standard deviation (SD), and the obviously skewed or unknown distributed variables were expressed as median and interquartile range (IQR). Categorical variables were presented as count (percentage). Comparison of clinical features between ARDS‐sepsis patients and non‐ARDS‐sepsis patients was determined by Student's *t* test, chi‐square test, or Wilcoxon rank sum test. Multiple comparisons of miR‐125a and miR‐125b relative expressions among HCs, ARDS‐sepsis patients and non‐ARDS‐sepsis patients were performed by Dunn's multiple comparison test. Comparisons of miR‐125a and miR‐125b relative expressions between survivors and deaths were analyzed by Wilcoxon rank sum test. Correlations of miR‐125a and miR‐125b relative expressions with variables were determined by Spearman's rank correlation test or Wilcoxon rank sum test. The factors predicting ARDS risk in sepsis patients were analyzed by univariate and multivariate logistic regression model. The performances of miR‐125a and miR‐125b relative expressions in predicting sepsis patients' ARDS risk were evaluated using receiver operating characteristic (ROC) curves and the area under the curve (AUC) with 95% confidence interval (CI). Statistical analyses were performed using SPSS 24.0 software (IBM), and figures were made using GraphPad Prism 7.01 software (GraphPad Software). *P* value <.05 was considered significant.

## 3 RESULTS

### 3.1 Comparison of characteristic between ARDS‐sepsis patients and non‐ARDS‐sepsis patients

The mean age of total sepsis patients was 56.9 ± 10.3 years, and there were 52 females and 98 males (Table 1). The mean APACHE II score and SOFA score were 16.3 ± 6.3 and 7.3 ± 3.2, respectively. Total sepsis patients (N = 150) were divided into ARDS‐sepsis patients (n = 41) and non‐ARDS‐sepsis patients (n = 109). The mean age (*P* = .006), percentage of patients smoking (*P* = .021), the percentage of patients with COPD (*P* = .004), CRP (*P* = .001), APACHE II score (*P* < .001), and SOFA score (*P* = .038) were increased in ARDS‐sepsis patients compared with non‐ARDS‐sepsis patients. The primary infection between non‐ARDS‐sepsis patients and ARDS‐sepsis patients were different (*P* < .001). There were 31 (28.4%), 39 (35.8%), 20 (18.4%), 8 (7.3%), 3 (2.8%), and 8 (7.3) non‐ARDS‐sepsis patients with lung infection, urinary tract infection, intra‐abdomen infection, skin and soft tissue infection, bone and joints infection, other infection, respectively, and meanwhile there were 30 (73.2%), 5 (12.2%), 3(7.3%), 2 (4.9%), 0 (0.0%), 1 (2.4%) ARDS‐sepsis patients with lung infection, urinary tract infection, intra‐abdomen infection, skin and soft tissue infection, bone and joints infection, and other infection, respectively. More detailed information of clinical characteristics was exhibited in Table 1.

**Table 1:** Clinical characteristics of sepsis patients

Items	Total sepsis patients (N = 150)	Non‐ARDS‐sepsis patients (n = 109)	ARDS‐sepsis patients (n = 41)	*P* value
Age (y), mean ± SD	56.9 ± 10.3	55.5 ± 10.0	60.6 ± 10.4	.006
Gender, No. (%)				
Female	52 (34.7)	38 (34.9)	14 (34.1)	.935
Male	98 (65.3)	71 (65.1)	27 (65.9)	
BMI (kg/m^2^), mean ± SD	22.8 ± 4.8	22.6 ± 4.8	23.5 ± 4.9	.299
Smoke, No. (%)	58 (38.7)	36 (33.0)	22 (53.7)	.021
Chronic comorbidities, No. (%)				
COPD	33 (15.3)	11 (10.1)	12 (29.3)	.004
Cardiomyopathy	49 (32.7)	35 (32.1)	14 (34.1)	.813
Chronic kidney failure	18 (12.0)	11 (10.1)	7 (17.1)	.241
Cirrhosis	28 (18.7)	22 (20.2)	6 (14.6)	.437
Primary infection, No. (%)				
Lung infection	61 (40.7)	31 (28.4)	30 (73.2)	<.001
Urinary tract infection	44 (29.3)	39 (35.8)	5 (12.2)	
Intra‐abdomen infection	23 (15.3)	20 (18.4)	3 (7.3)	
Skin and soft tissue infection	10 (6.7)	8 (7.3)	2 (4.9)	
Bone and joints infection	3 (2.0)	3 (2.8)	0 (0.0)	
Other infection	9 (6.0)	8 (7.3)	1 (2.4)	
Laboratory indexes, median (IQR)				
Scr (mg/dL)	1.7 (1.2‐2.4)	1.6 (1.1‐2.2)	1.8 (1.3‐3.1)	.141
Albumin (g/L)	27.2 (21.6‐36.8)	27.2 (21.7‐36.7)	27.8 (20.6‐38.3)	.655
WBC (×10^9^/L)	14.0 (2.9‐28.1)	12.5 (2.9‐26.9)	19.2 (3.2‐30.1)	.257
CRP (mg/L)	98.5 (52.7‐128.8)	83.7 (52.1‐121.0)	131.2 (72.1‐217.1)	.001
APACHE II score, mean ± SD	16.3 ± 6.3	15.2 ± 6.1	19.3 ± 5.9	<.001
SOFA score, mean ± SD	7.3 ± 3.2	6.9 ± 3.2	8.2 ± 3.0	.038

Note

Comparison was determined by Student's *t* test, chi‐square test, or Wilcoxon rank sum test.

Abbreviations: APACHE II score, acute pathologic and chronic health evaluation II score; ARDS, acute respiratory distress syndrome; BMI, body mass index; COPD, chronic obstructive pulmonary disease; CRP, C‐reactive protein; IQR, interquartile range; Scr, serum creatinine; SD, standard deviation; SOFA score, sequential organ failure assessment score; WBC, white blood cell.

### 3.2 Comparison of miR‐125a and miR‐125b among HCs, non‐ARDS‐sepsis patients, and ARDS‐sepsis patients

**Figure 1:**
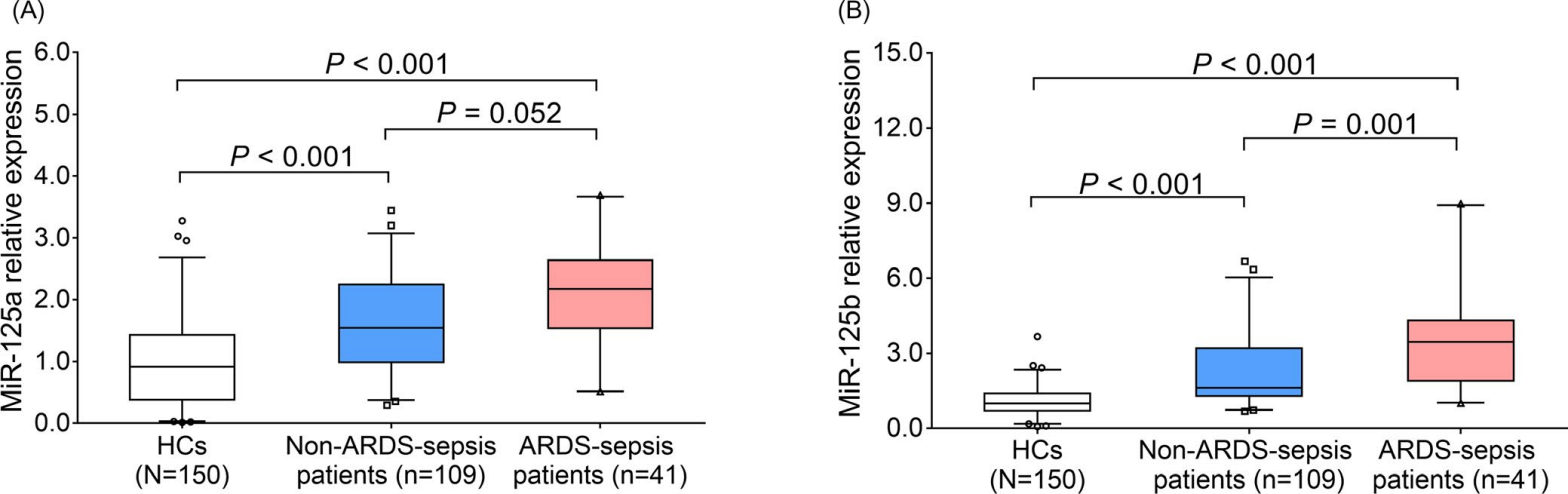
MiR‐125a and miR‐125b expressions. Comparison of miR‐125a between ARDS‐sepsis patients and non‐ARDS‐sepsis patients, between ARDS‐sepsis patients and HCs, and between non‐ARDS‐sepsis patients and HCs (A). Comparison of miR‐125b between ARDS‐sepsis patients and non‐ARDS‐sepsis patients, between ARDS‐sepsis patients and HCs, and between non‐ARDS‐sepsis patients and HCs (B). Multiple comparisons of miR‐125a and miR‐125b relative expressions among ARDS‐sepsis patients, non‐ARDS‐sepsis patients, and HCs were performed by Dunn's multiple comparison test. *P* value <.05 was considered significant. HC, healthy control; ARDS, acute respiratory distress syndrome; miR‐125a, microRNA‐125a; miR‐125b, microRNA‐125b

MiR‐125a and miR‐125b relative expression was increased in ARDS‐sepsis patients (both *P* < .001)/non‐ARDS‐sepsis patients (both *P* < .001) compared with HCs, and miR‐125b was also elevated in ARDS‐sepsis patients compared with non‐ARDS‐sepsis patients (*P* = .001), while miR‐125a was numerically elevated in ARDS‐sepsis patients compared with non‐ARDS‐sepsis without statistical significance (*P* = .052) (Figure 1A,B).

### 3.3 The values of miR‐125a and miR‐125b in predicting ARDS risk in sepsis patients

**Figure 2:**
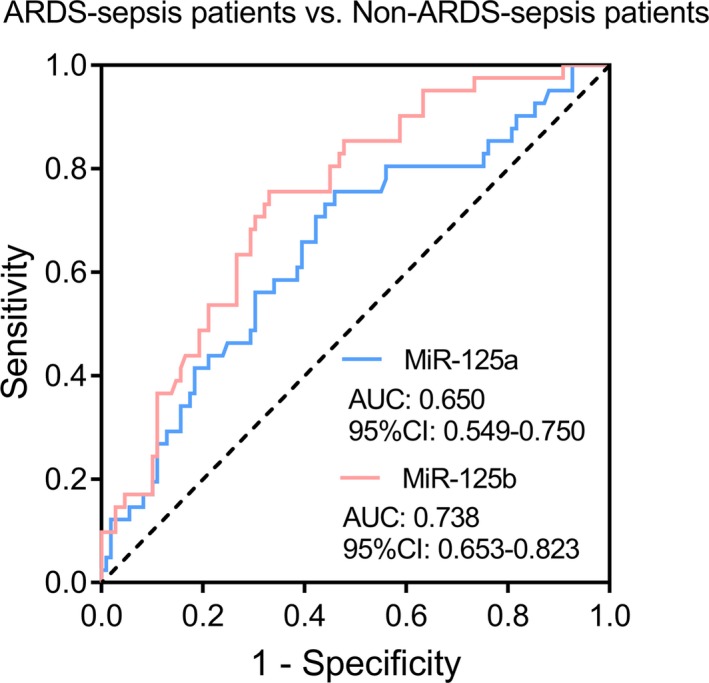
Predictive value of miR‐125a and miR‐125b expressions for ARDS risk. The performances of miR‐125a and miR‐125b relative expressions in predicting sepsis patients' ARDS risk were evaluated using ROC curves and the AUC with 95% CI. ARDS, acute respiratory distress syndrome; miR‐125a, microRNA‐125a; miR‐125b, microRNA‐125b; ROC, receiver operating characteristic; CI, confidence interval; AUC, area under the curve

Receiver operating characteristic curve presented that miR‐125a could distinguish ARDS‐sepsis patients from non‐ARDS‐sepsis patients to some extent with AUC of 0.650 (95%CI: 0.549‐0.750), and miR‐125b was of relatively good value in differentiating ARDS‐sepsis patients from non‐ARDS‐sepsis patients with AUC of 0.739 (95%CI: 0.653‐0.823) (Figure 2).

### 3.4 Factors predicting ARDS risk in sepsis patients

Univariate logistic regression analysis exhibited that miR‐125a (OR = 1.952, *P* = .006), miR‐125b (OR = 1.621, *P* < .001), age (OR = 1.052, *P* = .008), smoke (OR = 2.348, *P* = .022), COPD (OR = 3.687, *P* = .005), CRP (OR = 1.012, *P* < .001), APACHE II score (OR = 1.113, *P* = .001), and SOFA score (OR = 1.126, *P* = .040) was positively associated with ARDS risk in sepsis patients (Table 2). Multivariate logistic regression analysis revealed that both miR‐125a and miR‐125b failed to predict ARDS risk independently, while only COPD (OR = 5.762, *P* = .010), CRP (OR = 1.010, *P* = .014), and APACHE II score (OR = 1.122, *P* = .042) were independent predictive factors for higher ARDS risk in sepsis patients (Table 2).

**Table 2:** Univariate and multivariate logistic regression model analyses of factors predicting ARDS risk in sepsis patients

Items	Univariate logistic regression		Multivariate logistic regression	
	*P* value	OR (95%CI)	*P* value	OR (95%CI)
MiR‐125a	.006	1.952 (1.217‐3.131)	.470	1.283 (0.652‐2.525)
MiR‐125b	<.001	1.621 (1.281‐2.053)	.429	1.169 (0.793‐1.724)
Age	.008	1.052 (1.013‐1.093)	.187	1.036 (0.983‐1.093)
Male	.935	1.032 (0.485‐2.199)	.742	0.840 (0.297‐2.373)
BMI	.298	1.040 (0.966‐1.119)	.415	1.043 (0.943‐1.153)
Smoke	.022	2.348 (1.129‐4.883)	.106	2.239 (0.841‐5.958)
COPD	.005	3.687 (1.473‐9.223)	.010	5.762 (1.507‐22.028)
Cardiomyopathy	.813	1.096 (0.512‐2.345)	.740	1.211 (0.390‐3.764)
Chronic kidney failure	.246	1.834 (0.658‐5.111)	.720	1.291 (0.319‐5.217)
Cirrhosis	.439	0.678 (0.253‐1.814)	.172	0.393 (0.103‐1.499)
Scr	.152	1.208 (0.933‐1.566)	.648	0.910 (0.608‐1.364)
Albumin	.608	0.990 (0.951‐1.030)	.237	0.958 (0.892‐1.029)
WBC	.177	1.016 (0.993‐1.041)	.994	1.000 (0.959‐1.043)
CRP	<.001	1.012 (1.006‐1.018)	.014	1.010 (1.002‐1.019)
APACHE II score	.001	1.113 (1.046‐1.184)	.042	1.122 (1.004‐1.254)
SOFA score	.040	1.126 (1.005‐1.262)	.383	0.917 (0.754‐1.115)

Abbreviations: APACHE II score, acute pathologic and chronic health evaluation II score; ARDS, acute respiratory distress syndrome; BMI, body mass index; CI, confidence interval; COPD, chronic obstructive pulmonary disease; CRP, C‐reactive protein; miR, microRNA; OR, odds ratio; Scr, serum creatinine; SOFA score, sequential organ failure assessment score; WBC, white blood cell.

### 3.5 Correlation of miR‐125a and miR‐125b with clinical characteristics in sepsis patients

For continuous variables, miR‐125a relative expression was positively associated with Scr (*r* = .207, *P* = .011), APACHE II score (*r* = .287, *P* < .001), and SOFA score (*r* = .209, *P* = .010) (Table 3). MiR‐125b was positively associated with Scr (*r* = .238, *P* = .003), CRP (*r* = .414, *P* < .001), APACHE II score (*r* = .351, *P* < .001), and SOFA score (*r* = .278, *P* = .001). As for the categorical variables, miR‐125b was associated with COPD (*P* = .048) (Table 4).

**Table 3:** Correlation of miR‐125a and miR‐125b relative expressions with continuous variables in sepsis patients

Items	MiR‐125a relative expression		MiR‐125b relative expression	
	*P* value	Correlation coefficient (*r*)	*P* value	Correlation coefficient (*r*)
Age	.985	−.002	.374	.073
BMI	.756	.026	.810	−.020
Scr	.011	.207	.003	.238
Albumin	.277	−.089	.993	.001
WBC	.601	.043	.605	.043
CRP	.073	.147	<.001	.414
APACHE II score	<.001	.287	<.001	.351
SOFA score	.010	.209	.001	.278

Note

Correlation was determined by Spearman's rank correlation test.

Abbreviations: APACHE II score, acute pathologic and chronic health evaluation II score; BMI, body mass index; CRP, C‐reactive protein; miR, microRNA; Scr, serum creatinine; SOFA score, sequential organ failure assessment score; WBC, white blood cell.

**Table 4:** Correlation of miR‐125a and miR‐125b relative expressions with categorical variables in sepsis patients

Items	MiR‐125a relative expression		MiR‐125b relative expression	
	Median (IQR)	*P* value	Median (IQR)	*P* value
Gender				
Male	1.764 (1.075‐2.406)	.400	1.863 (1.372‐3.889)	.634
Female	1.797 (0.847‐2.336)		1.689 (1.453‐3.054)	
Smoke				
No	1.874 (1.105‐2.407)	.343	1.781 (1.419‐3.675)	.796
Yes	1.664 (0.947‐2.332)		1.773 (1.363‐3.893)	
COPD				
No	1.700 (1.079‐2.372)	.701	1.668 (1.343‐3.354)	.048
Yes	2.006 (0.953‐2.394)		2.888 (1.505‐3.941)	
Cardiomyopathy				
No	1.800 (0.959‐2.383)	.583	1.811 (1.348‐3.791)	.746
Yes	1.682 (1.118‐2.501)		1.570 (1.469‐3.833)	
Chronic kidney failure				
No	1.771 (1.027‐2.402)	.910	1.723 (1.419‐3.694)	.779
Yes	1.826 (1.060‐2.162)		2.936 (1.071‐4.149)	
Cirrhosis				
No	1.781 (1.075‐2.359)	.915	1.863 (1.331‐3.826)	.767
Yes	1.795 (0.976‐2.402)		1.603 (1.525‐3.282)	

Note

Correlations were determined by Wilcoxon rank sum test.

Abbreviatios: COPD, chronic obstructive pulmonary disease; miR, microRNA.

### 3.6 Comparison of miR‐125a and miR‐125b between survivors and deaths in sepsis patients

**Figure 3:**
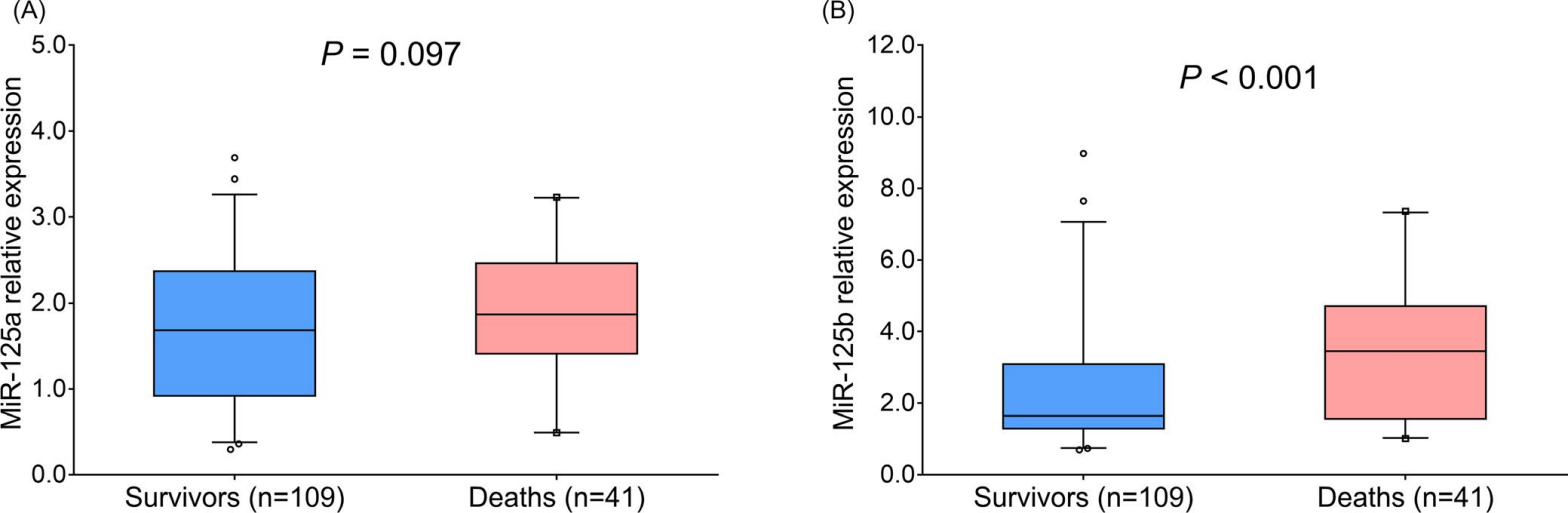
MiR‐125a and miR‐125b expressions in survivors and deaths. Comparison of miR‐125a (A) and miR‐125b (B) between survivors and deaths in sepsis patients. Comparison of miR‐125a and miR‐125b relative expressions between survivors and deaths were analyzed by Wilcoxon rank sum test. *P* value <.05 was considered significant. ARDS, acute respiratory distress syndrome; miR‐125a, microRNA‐125a; miR‐125b, microRNA‐125b

There was no difference of miR‐125a relative expression between survivors (1.682 [0.921‐2.372]) and deaths (1.868 [1.412‐2.465]) (*P* = .097) in sepsis patients (Figure 3A). However, miR‐125b relative expression in deaths (3.450 [1.563‐4.709]) was increased compared with survivors (1.646 [1.295‐3.091]) (*P* < .001) in sepsis patients (Figure 3B).

## 4 DISCUSSION

In the present study, we found that in sepsis patients (a) miR‐125a and miR‐125b could both predict ARDS risk, and miR‐125b was of increased value in predicting ARDS risk compared with miR‐125a numerically; (b) miR‐125a was associated with renal dysfunction, higher disease severity but not inflammation, and miR‐125b was correlated with renal dysfunction, COPD, increased inflammation, and higher disease severity; (c) miR‐125b but not miR‐125a was upregulated in deaths compared with survivors.

MiR‐125a and miR‐125b are considered as important factors in the mechanisms of several immune and inflammation responses and play crucial roles in many different cellular processes including cell differentiation, proliferation, and apoptosis.^8^, ^9^, ^18^, ^19^ For example, miR‐125a is shown to regulate maturation of neutrophils by targeting the major suppressor of G‐CSF signaling and neutrophils SOCS3 and serve as an important regulator of granulopoiesis in lung diseases.^20^ As for miR‐125b, it is elevated in patients with rheumatoid arthritis compared with HCs and the increased expression of miR‐125b has been found in patients with other chronic inflammatory arthritis.^21^ In another study, miR‐125b presents association with sepsis‐induced cardiac inflammation and dysfunction via p38 MARK/ NFκB signaling pathway.^22^ A recent study suggests the correlation of miR‐125b with acute exacerbations of COPD as well as the expressions of inflammatory.^19^ Considering the association of miR‐125a and miR‐125b with lung injuries and inflammation/immune regulation, we hypothesized that miR‐125a and miR‐125b might have potential for predicting ARDS risk in sepsis patients. In this study, we found that miR‐125a and miR‐125b relative expressions were increased in ARDS‐sepsis patients compared with non‐ARDS‐sepsis patients and HCs, and miR‐125a as well as miR‐125b could predict ARDS risk in sepsis patients. MiR‐125b was of increased value in predicting ARDS risk compared with miR‐125a numerically. The possible reasons might include that miR‐125a and miR‐125b might act as potent regulators of the inflammation pathways (such as NF‐κB signaling), which contributed to activation of inflammation cells and release of inflammatory cytokines, and meanwhile, miR‐125b was shown to serve as a hypoxia‐related miRNA and induce the injury of lung tissue via regulating apoptosis of airway epithelial cells, which combinedly led to increased lung barrier function and the development of lung injury. This was also supported by one of our findings that miR‐125b was associated with COPD in sepsis patients. Thus, miR‐125a and miR‐125b were associated with higher risk of ARDS in sepsis patients.^19^ As for the numerically increased predictive value of miR‐125b compared with miR‐125a in ARDS risk, miR‐125b was associated with inflammation, complicated diseases of lung (such as COPD), it might be due to that miR‐125b rather than miR‐125a was associated with inflammation and complicated diseases of lung (such as COPD) in sepsis patients. It was also interesting to note that miR‐125 and miR‐125b were not independent predictive factors for higher ARDS risk. This could be explained by that miR‐125a as well as miR‐125b might interact with other clinicopathological features, such as COPD, higher level of inflammation, and advanced disease severity, to affect the ARDS risk. Hence, miR‐125a and miR‐125b were not independent predictive factors for ARDS risk in sepsis patients. This might be supported by one of our results that miR‐125a and miR‐125b were both positively associated with SOFA score and APACHE II score and that miR‐125b was positively correlated with COPD; meanwhile, these three factors were shown to be independent predictive factors for ARDS risk.

Several studies indicate the involvement of miR‐125a and miR‐125b in inflammatory pathologies and miR‐125a and miR‐125b have ability of regulating the secretion of proinflammation chemokines.^19^, ^21^, ^22^ For example, miR‐125b is associated with human osteoarthritis and regulate the secretion of IL‐1β and TNF‐α.^23^ In another study, miR‐125b is associated with increased inflammatory cytokines, including TNF‐α, IL‐8, and LTB‐4, and acts as a critical role in the initiation and progression of acute exacerbation COPD.^19^ A recent study also reveals the correlation of miR‐125a with CRP, IL‐17, TNF‐α, and Crohn's disease activity index in patients with active Crohn's disease.^24^ In addition, the association of miR‐125a and miR‐125b with oxygen and glucose deprivation via regulating reactive oxygen species (ROS) level is reported in several organs, such as lung and kidney.^13^, ^14^ In our present study, we found that miR‐125a was positively associated with Scr, APACHE II score, and SOFA score, and miR‐125b was positively associated with Scr, CRP, APACHE II score, SOFA score, and COPD in sepsis patients. The possible reasons might include that (a) according to the previous study, miR‐125b might promote oxidative damage as well as enhance the secretion of inflammation factors in kidney and lung, leading to renal dysfunction and increased COPD risk, which contributed to higher disease severity in sepsis patients. (b) Considering that miR‐125a was of both anti‐inflammatory and proinflammatory effects, thus there was no association of miR‐125a with inflammation in sepsis patients. MiR‐125a might contribute to the increased disease severity via causing renal dysfunction in sepsis patients. However, the underlying mechanism of miR‐125a and miR‐125b in sepsis needed further exploration in cellular experiments.

Considering the findings that miR‐125a and miR‐125b were associated with higher inflammatory level and advanced disease severity in sepsis patients, we further explored the association of miR‐125a and miR‐125b with survival profiles in sepsis patients. We observed that miR‐125b but not miR‐125a was upregulated in deaths compared with survivors in sepsis patients. The possible explanations might be that (a) according to the previous findings, miR‐125a was associated with APACHE II score and SOFA score, and there was no association of miR‐125a with systematic inflammation and lung injuries, therefore, miR‐125a lacked power to predict mortality in sepsis patients. (b) as for miR‐125b, it was not only associated with disease severity, but also correlated with systematic inflammation and multiple organ dysfunction (such as renal and lung), which suggested the potential value of miR‐125b in predicting mortality in sepsis patients.

There were several limitations in our study. (a) The sample size of our study was relatively low, thus study with a larger sample size was needed for validation in the future. (b) The sample size was relatively small to achieve strong statistical power; thus, a large sample size was needed for validation. (c) The underlying mechanism of miR‐125a and miR‐125b was not included in our present study, which needed further exploration.

MiR‐125a and miR‐125b correlates with increased ARDS risk and disease severity of sepsis, while only miR‐125b associates with elevated systemic inflammation and raised mortality in sepsis patients. These imply miR‐125b might be a potential marker for the management of sepsis.
